# Mental health policy process: a comparative study of Ghana, South Africa, Uganda and Zambia

**DOI:** 10.1186/1752-4458-4-24

**Published:** 2010-08-02

**Authors:** Maye A Omar, Andrew T Green, Philippa K Bird, Tolib Mirzoev, Alan J Flisher, Fred Kigozi, Crick Lund, Jason Mwanza, Angela L Ofori-Atta

**Affiliations:** 1 Nuffield Centre for International Health and Development, Leeds Institute of Health Sciences, University of Leeds, 101 Clarendon Road, Leeds LS2 9LJ, UK; 2 Department of Psychiatry and Mental Health, University of Cape Town, South Africa; 3 Butabika National Referral Mental Hospital and Department of Psychiatry, Makarere University, Kampala, Uganda; 4 Department of Social Development Studies Division of Sociology University of Zambia, Zambia; 5 University of Ghana Medical School, Accra, Ghana

## Abstract

**Background:**

Mental illnesses are increasingly recognised as a leading cause of disability worldwide, yet many countries lack a mental health policy or have an outdated, inappropriate policy. This paper explores the development of appropriate mental health policies and their effective implementation. It reports comparative findings on the processes for developing and implementing mental health policies in Ghana, South Africa, Uganda and Zambia as part of the Mental Health and Poverty Project.

**Methods:**

The study countries and respondents were purposively selected to represent different levels of mental health policy and system development to allow comparative analysis of the factors underlying the different forms of mental health policy development and implementation. Data were collected using semi-structured interviews and document analysis. Data analysis was guided by conceptual framework that was developed for this purpose. A framework approach to analysis was used, incorporating themes that emerged from the data and from the conceptual framework.

**Results:**

Mental health policies in Ghana, South Africa, Uganda and Zambia are weak, in draft form or non-existent. Mental health remained low on the policy agenda due to stigma and a lack of information, as well as low prioritisation by donors, low political priority and grassroots demand. Progress with mental health policy development varied and respondents noted a lack of consultation and insufficient evidence to inform policy development. Furthermore, policies were poorly implemented, due to factors including insufficient dissemination and operationalisation of policies and a lack of resources.

**Conclusions:**

Mental health policy processes in all four countries were inadequate, leading to either weak or non-existent policies, with an impact on mental health services. Recommendations are provided to strengthen mental health policy processes in these and other African countries.

## Background

Mental health is a corner stone of health [1]. Mental illnesses are increasingly recognised as a leading cause of disability worldwide [2], with neuro-psychiatric conditions accounting for 14% of the global disease burden [2]. Depression currently affects over 450 million people, most of them poor and from developing countries [3]. By 2020 unipolar depressive diseases will be the second most important cause of disability [4].

However, despite the growing burden of mental illness and the resultant level of suffering for individuals and society, efforts to address it are unsatisfactory. This is particularly true in developing countries due to low budgetary resources [5], presence of competing and conflicting health system needs, scarcity of mental health personnel, and the stigma involved in seeking psychiatric help [6]. For example, of the 24 million people with schizophrenia worldwide, half do not receive appropriate care; 90% are in developing countries [7-9].

Mental health policies signal a government's intent to address the mental health needs of its citizens. However, many countries either lack such a policy, or have non-operational, inappropriate policy. For example, 53% of African countries have a mental health policy, and many of these are outdated [10].

The bulk of health policy research has focused on policy *content*, often, for example, evaluating technical appropriateness. Only relatively recently have researchers turned their attention to the *processes *of health policy development and implementation. The few existing studies of mental health policy in Africa have tended to focus on individual countries [11] and no comparative studies have been conducted.

This paper explores the factors that underpin the development of appropriate mental health policies and their effective implementation. It reports comparative findings on the processes for developing and implementing mental health policies in Ghana, South Africa, Uganda and Zambia. This study forms part of the Mental Health and Poverty Project, which aims to develop, implement and evaluate mental health policies in these countries [12]. Findings from individual study countries have been reported elsewhere [13-17].

## Methods

**Figure 1:**
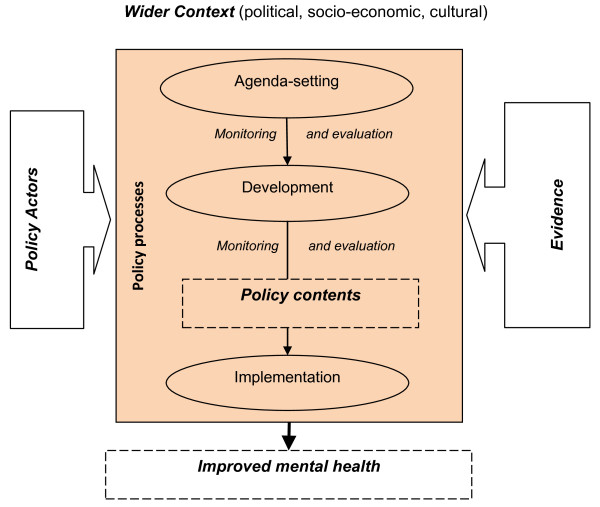
**Conceptual framework for comparison of mental health policy processes in four African countries**. Adapted from: [12,14,15].

Conceptual models, theories, and frameworks can provide tools to describe, understand and explain health policy processes [18]. Our conceptual framework was derived from various other frameworks (see Figure 1).

It distinguishes various elements. *Health policy processes *(*agenda-setting, development *and *implementation) *are the main focus of the current paper. Different *policy actors*, either individually or as part of wider networks, engage in these processes to varying degrees. Involvement of actors can be direct or indirect and is influenced by their agendas, values, ideologies and relative powers. Different types of *evidence *exist within the health system and may be used to inform health policy processes. The wider *contextual factors *such as degree of political support or cultural attitudes towards mental health can facilitate or constrain policy processes. The *policy contents *(outside the scope of the current study) may lead to changes in processes as well as a range of effects on health or the health care system.

The study countries were purposively selected to represent different levels of mental health policy and system development, allowing comparison of the factors underlying the different forms and attributes of mental health policy development and implementation.

We collected qualitative data using semi-structured interviews in Ghana (58), South Africa (64), Uganda (60) and Zambia (65). The research methods were developed collaboratively by the research consortium, then adapted to fit the local context by the country research teams. The semi-structured interview respondents were selected purposively and through snowballing to include national and regional level groups with experience or interest in mental health policy processes. This included policymakers, programme managers, media, medical professional associations, traditional healer unions, mental health user groups and other relevant sectors including prisons, justice, social development, housing and education. We collected and reviewed documents identified in literature searches and those suggested by respondents.

Analysis of primary data was done in each study country using NVivo 7. A framework approach to analysis [19], was used, incorporating themes that emerged from the data and from our conceptual framework. The comparative cross-country analysis reported here was conducted by University of Leeds team, using the country reports as the information source. To ensure that comparative analysis was systematic, we developed a matrix of key themes and findings from each country. Comparative findings were validated with researchers in the country teams.

## Results

We first report findings on the wider country contexts. We then discuss each policy process stage, taking into account policy actors and use of evidence. We then consider the role of contextual factors in explaining similarities and differences between the country processes.

### Context

Apart from South Africa, a middle-income country, Ghana, Uganda and Zambia were low-income countries. However, all countries had high levels of poverty and inequality [20,21]

#### Structure and financing of the health system

The countries had different forms of decentralised health systems. The Ghana Health Service maintains a relatively high level of control for policy implementation through the Ghana Health Service Council. However, some decision-making power and health sector management has been delegated to regions, including providing supervision and management support to districts and sub-districts. In Uganda, there has been devolution to the district level, with local governments playing a significant role in political and health decision-making. The Zambian health system is deconcentrated to regional and district health structures. In South Africa devolution has created a semi-federal structure with provinces having substantial authority regarding health planning and budget allocations.

In all countries there were low levels of financial and human resources for health (Table 1). External support comprises 20-40% of health expenditure in Ghana, Uganda and Zambia (all of which have implemented sector-wide approaches (SWAps) but is negligible in South Africa. Financial resources for mental health were generally considered inadequate. Ghana devoted the largest proportion (approximately 6%) of the government health budget to mental health. However, Zambia devoted only 0.4%. In Uganda, government mental health expenditure is normally around 1% but was 4% at the time of the study due to an African Development Bank loan. South Africa experienced wide variation between provinces in the proportion of the health budget allocated to mental health (from 1% in the Northern Cape to 8% in Mpumalanga). Where mental health was part of primary care with integrated budgets, there was uncertainty about the proportion devoted to mental health.

**Table 1:** Health system and mental health expenditure

	Total expenditure on health as % GDP *	Per capita total expenditure on health (PPP int. $) *	External resources to health as % of total expenditure on health *	Mental health budget as % total government health budget **
Ghana	5.1	76	22.6	6

South Africa	8.0	715	0.9	1-8% (Provincial variation)

Uganda	7.0	71	31.2	4

Zambia	3.9	79	38.1	0.4%

Sources: *[35]; **[8]

#### The mental health care system

Mental health services are predominantly government-provided through dedicated psychiatric hospitals, psychiatric units and clinics. The private health care sector plays a limited role in provision of mental health services, although many people with mental illnesses turn to spiritual or traditional healers for help. Few NGOs work directly on mental health, but a large number provide mental health services as part of other programmes, for example counselling in HIV programmes. South Africa is an exception, where the South African Federation for Mental Health (a national NGO) provides an extensive network of mental health services, including half of all community-based residential facilities.

#### Priority problems and position of mental health

Given the low level of resources for health and high burden of disease, mental health has to compete for resources. Mental health is considered a low priority relative to other social, economic and health problems. Poverty eradication is the main political priority in Uganda, with all government interventions feeding into this strategy; while in South Africa the focus is on development and service provision for those disadvantaged and discriminated against by apartheid. Communicable diseases were considered the highest priority conditions in all countries, in particular HIV/AIDS in South Africa and malaria in Ghana.

#### Public attitudes to mental illness

Respondents commented on the poor knowledge and understanding of mental illnesses among the general public. Mental illnesses were stigmatised, with sufferers and people working in mental health reporting negative attitudes towards them. Among the general public, mental illnesses were widely attributed to supernatural and spiritual causes; although some respondents suggested that mental illnesses were treatable.

### Current status of mental health policy

**Figure 2:**
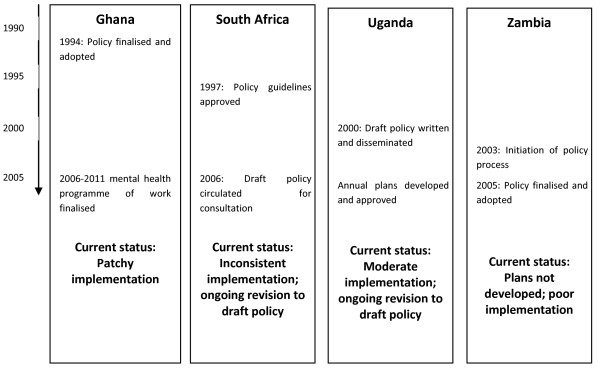
**Mental health policy and plan timeline**.

The countries were at different stages of the mental health policy process (Figure 2). Only Ghana and Zambia had an approved national mental health policy. Ghana finalised this in 1994 and a five-year mental health programme of work was developed for implementation. The Zambian policy was finalised in 2005 but mental health plans had not been developed and implementation was poor.

South Africa and Uganda had draft policies undergoing further revisions. In South Africa, policy *guidelines *were approved in 1997. However, a formal mental health policy has not yet been adopted, although a draft had been circulated for comment in 2006. The guidelines have been inconsistently implemented at the provincial level. In Uganda there was a similar situation. The policy has remained at a draft stage since 2000, although there was some implementation through annual mental health plans.

### Mental health policy processes

#### Agenda setting

Three avenues to policy initiation were identified by a Ugandan senior policymaker. Policies could be demand driven, following urgent pressing social problems; they could emerge to address weaknesses identified in routine performance or assessment report; or could be initiated by political leaders to fulfil election pledges. In Zambia, one respondent suggested that issues get on the agenda as a result of complaints from general public or civil society organisations (CSOs), commenting:

*"usually the public and the other institutions stimulate us when they complain about something or certain aspects of an existing policy"*.

Given these potential avenues, CSOs and the general public could play a role in getting mental health on the agenda. However, despite the high mental illness burden, there was limited demand from the public for improved mental health policy. Stigma towards mental illness contributed to the low priority of mental health and minimised their role in agenda-setting. There was little evidence of an active role by CSOs in the advocacy of mental health issues. Even in South Africa, with several mental health service user groups, these groups were fragmented and tended to mainly provide support to people with mental illnesses rather than conduct policy advocacy. This is significantly different to the policy environment for HIV/AIDS in South Africa, and to some degree in the other countries, where civil society has an important advocacy role.

Mental health was low on the agenda of some policymakers, being a low political priority and poorly understood and valued by the public [22]. Furthermore, stigmatization of mental illness among policymakers may deter them from taking up mental health issues, as reported in South Africa. The invisibility of mental illness and lack of tangible outcomes from interventions were also cited as reasons for low awareness of mental health among policy-makers.

The issues on the policy agenda also reflected data availability. Lack of data was a general problem, attributed to the lack of mental health indicators in the Health Management Information Systems (HMIS) and the inability of health workers to identify and record mentally ill patients due to inadequate clinical skills and knowledge. The low utilization of health services by mentally ill patients also affects accuracy of incidence data. In Uganda, for example, with minimal data collected on mental illness, it was impossible to quantify the disease burden. The small number of cases reported in the HMIS was used to justify the low consideration of mental health.

International evidence was cited as influential in some countries. For example, in Uganda, the 2001 World Health Report helped to raise the profile of mental health. This provided evidence to inform the policy and triggered the development of the mental health policy. Health issues with international targets such as MDGs became the highest national priority issues; however there is no mental health MDG. Donor support to mental health was generally low and donors prioritized other health conditions. Comparing mental illness with HIV/AIDS and malaria, health workers felt that resources were available for other conditions, but not for mental health. One Ugandan nurse criticised donors' priorities:

"...*and whoever comes in just looks at a proposal for AIDS, malaria...among 100 proposals for donors,, there may be 1 for mental health. So, somebody has to look at what is selling*"

In summary, issues could get on the policy agenda for various reasons, including prioritisation by donors, political agenda or grassroots demand. However stigma and a lack of mental health information act as a significant barrier.

#### Policy development

The time taken for developing policy differed (Figure 2). It was lengthy in South Africa and Uganda, with delays during policy drafting and formal approval. This may, in part, be due to bureaucratic hurdles, poorly informed policymakers, lack of ready evidence and stigma of mental illness. In contrast, in Zambia it took just 2 years to develop the policy. This was seen to be the result of extensive lobbying by the Mental Health Unit.

Respondents described an 'ideal' or formal process for developing policy. This was perceived to be one that starts with a situational analysis, followed by identification of the needs and problems to be addressed and then setting out strategies to address them. They saw a bottom-up approach as most appropriate; however many expressed uncertainties with regard to how policy development should start and the levels at which policies have to be approved. In South Africa, eight steps for developing policy were identified, including approval by different government bodies and development of detailed guidance documents (e.g. budgets and plans). It was, however, reported that many policies, including the mental health policy, do not go through this process, largely due to limited resources. In particular, respondents complained that there had been insufficient stakeholder consultation.

In all four countries, the mental health policy process was led by the health ministry. Consultation focused largely on government health employees. In Ghana, for example, consultation included the psychiatric hospital medical directors, Ministry of Health Departments and the health research directorate. Such consultation was largely at the national level. Regional and district level respondents in Ghana and Uganda reported that policymaking was carried out by a few senior managers at national level with little consultation of lower level workers.

Consultation outside the health sector was not reported. As a Zambian Prisons Department respondent said:

"Mental health can only be integrated in our system if you people invite us in your activities. I am not just saying you invite the prisons alone, but all the stakeholders who seem to be playing a role in the provision of mental health services."

The majority of respondents emphasised the importance of involving users, potential users and those who benefit from interventions. This was due both to their knowledge about needs and priorities, and their right to be involved. In Zambia there was strong emphasis by many respondents on the need to involve advocacy groups when developing policies. South African respondents also raised the importance of promoting users to change attitudes to mental illness. A mental health user from Uganda, citing the disability movement slogan, "*nothing about us without us*", noted:

"They [mentally ill] have a right to self determination. Without their involvement, they would disown the Policy and Act, considering them to be for the professionals who developed them." 

Despite these generally positive opinions on involving users, mental health users were largely neglected in the consultation process. A respondent from the Ghana health ministry suggested that stigma was a barrier to involvement. Although there were mental health user groups in each country, they tended to be fragmented with limited organisation through networks or partnerships. Some respondents were also unsure or negative about the involvement of users in developing policy, voicing concerns about the large number of users and practical challenges.

Lastly, actors in certain geographical areas or ethnic groups were reported to be excluded from policy development. In Ghana there was no consultation in rural areas or the north.

In South Africa respondents suggested a need to take account of the wide cultural and linguistic diversity in consultation. CSO respondents suggested that there should have been a series of consultations with CSOs, mental health service users, traditional and spiritual healers, religious leaders and international organizations and donors. The above suggests absence of a clear stakeholder involvement framework

Information to support policymaking was either lacking (Ghana and Zambia) or inadequate and inappropriate (South Africa and Uganda). For example, in Uganda, it was reported that no specific needs assessment was done to inform the draft mental health policy. In Zambia a respondent noted:

"But with the data that we have, we can't go anywhere in policy and practice... You can imagine they couldn't come out with the number of psychiatrists that we have in the country, not even psychologists you see? 

Senior official, Ministry of Health, Zambia

In summary, progress with mental health policy development varied between the countries. In particular, respondents noted a lack of consultation and insufficient evidence to inform policy development.

#### Policy Implementation

The third stage of the policy processes studied was implementation. At one level, implementation of policies requires the prior existence of such policies - yet these are not formally approved in either Uganda or South Africa. However, the existence of an advanced draft in Uganda and the Mental Health Care Act (2002) and policy guidelines in South Africa were seen by respondents as being de facto policies and providing a legitimacy to implement these draft policies. This section, therefore, analyses the perceived policy implementation in the four countries, covering both approved and de-facto policies.

Policy implementation was considered generally poor by respondents who identified a number of causal factors.

Translation of policies into strategic plans (*policy operationalisation**) ***appeared to be a challenging but important step for effective implementation. Where the policy was translated into plans, such as Uganda, implementation was reported to be enhanced.

In Zambia, although the policy had been approved, plans for implementation had not been developed. On the other hand, despite the lack of policy, South Africa has a well developed Mental Health Care Act and the Ministry of Health has developed provincial level policy implementation guidelines. Because of the decentralised system in the country the national Department of Health (DoH) is tasked to develop policies, and the provincial DoH is meant to implement these policies through provincial level strategic plans. However, with the lack of an officially endorsed mental health policy, provincial planners have not had a clear and unambiguous message regarding their roles. In addition, mental health planners at provincial level are often very junior within provincial administrative hierarchies, and as a result lack the authority to effectively implement national policy. In the midst of such confusion caused by lack of clear roles and authority, implementation of policy guidelines was patchy.

Development of plans alone was not seen as sufficient for effective implementation and there seems to be a need for an appropriate coordinating body to oversee and lead implementation. In Ghana, despite the development of a five-year programme for mental health, implementation was weak. This was seen as partly the result of a lack of a unit responsible for mental health in the health ministry or its implementing wing, the Ghana Health Service. Mental health in Ghana is the responsibility of the chief psychiatrist who also oversees all three psychiatric hospitals, while heading the largest hospital in the capital city, with no institutional implementation support.

Many respondents perceived dissemination and communication of policy as important in implementation. However this seemed to occur inadequately. In South Africa, the approved policy guidelines were neither published nor circulated; in Ghana, few respondents were aware of the existence of policy documents. In Uganda others who *did *receive a copy of the draft policy had not read it; their explanation being that they had not been part of what they perceived as a top-down development process. This was echoed by respondents who felt that wide stakeholder involvement in policy development is a determinant for their ownership of policy implementation.

*"...if you have developed a policy from up there and you think it is good for me, and I have not contributed, then you should come down and assist me to implement. Because if I don't have a stake in the development of the policy, and you want me to have a stake in implementation, I may not value it the way you value it ... So, I think the stakeholders should be involved at all levels of policy; formulation, implementation, evaluation" *(Health Manager at District Level in Uganda)

Our findings suggest that the health system structure (particularly the form of decentralization) affected policy implementation through their implications on capacity and autonomy of local levels. In each country, implementation authority is devolved to regions, provinces and districts. However, low capacity is seen as a major problem. In South Africa, provincial mental health coordinators responsible for translating mental health policy guidelines into programmes were relatively junior in management structures, with little influence on resource allocation. In Ghana, respondents felt that the low level of regional autonomy was a cause of poor implementation; regional mental health coordinators lacked the capacity to plan and implement activities laid out in the policy and were not in a position to promote the prioritisation and resourcing of mental health services. In Uganda, although the draft mental health policy was translated into annual plans and budgets, the main challenge was the low level of the ring-fenced budget available to implement the policy.

Poor implementation may also stem from insufficient consideration of implementation during policy development. Ugandan district respondents reported a lack of commitment for implementation:

*"...The bad thing is that they have generated so many policies. People concentrate so much on developing the policies but they leave implementation to whom it may concern. They should put the same commitment in implementation. When you develop the policy, develop the implementation guidelines as well. So, there is a big gap there. The policies have been developed; but operationalisation of the policies is so limited" *(Uganda District Health Manager).

Lastly, policies may be overambitious in terms of the likely resources. Even in Ghana (with the largest proportion of its budget to mental health) respondents felt that mental health objectives and activities were simply not feasible, given the available level of resources.

## Discussion

Mental health policies in Ghana, South Africa, Uganda and Zambia are weak, in draft form or non-existent; furthermore they are poorly implemented. Mental health policies may not be implemented due to lack of feasible plans and inadequate resource commitments. Though all health services in the four countries are poorly resourced, mental health is a particularly neglected area. This appears to be the result of factors related to the wider context, roles and power of different policy actors and the policy processes themselves.

A number of contextual influences, both positive and negative, were found to be important. Mental health is not perceived as a social priority. Although the perception of, and attitudes towards, mental illness varied, there is generally poor knowledge and understanding of mental disorders among different stakeholders. This is partly due to the lack of routine information to inform mental health policy processes, combined with limited capacity to utilise available information. Furthermore, the available mental health evidence tends to focus narrowly on clinical aspects with less relevance for public health and policy issues [23]. This phenomenon is not new with evidence supply being acknowledged as one of the 'hindrances' to evidence-informed policymaking [24] and the quality of available evidence being increasingly questioned [25].

Amongst the public, mental illnesses are widely attributed to supernatural and spiritual causes. Stigma against mental illness, both generally in society and among policymakers and health professionals in particular, appears to be an important factor negatively affecting mental health policy processes. It affects the priority given to developing policies on mental health, and the subsequent assignment of resources. Stigma also affects people working for mental health, and deters others from joining. Thus stigma limits the capacity to bring mental health onto the policy agenda and subsequently limits the development and implementation of a policy.

Health policy processes are often weak and under-resourced. This is particularly evident within mental health, resulting in bureaucratic delays and poor use of any existing evidence. The lack of skills to operationalise policies into feasible programmes and plans as well as limited capacity to ensure adequate implementation were seen as key causes of poor implementation, and are themselves a reflection on the low priority given to mental health - a form of vicious cycle.

Decentralisation may also contribute to the fragmentation between policy development and policy implementation. Ineffective decentralisation can result in a lack of local capacity and expertise [26], which is particularly important for translation of centrally-developed policies into feasible local programmes and plans.

The importance of adequate involvement of various policy actors throughout the public policy process, either individually or as part of larger consortia or networks is increasingly understood [27,28]. Policy actors attempt to influence, and even control, policy decisions, particularly during the emergence, formulation and development phases. Their relative power is cited as a key determinant of actors' involvement in health policy processes [29-31]. However, mental health policy processes in these countries do not adequately consult key stakeholders including target groups and other key sectors. WHO [32] sets out an ideal framework which ought to embrace stakeholders with responsibility for funding, provision and regulation. It appears that not all of these were adequately involved.

Non-health departments, NGOs, user groups and professional organisations were frequently not consulted when developing mental health policies. Whilst advocacy from civil society can be a key driver of agenda-setting for other health issues, this was not the case for mental health. The perceived lack of an empowered civil society can hinder effective engagement of an important group in mental health policy processes, particularly at the development stage. The presence of a policy champion may help to promote policy processes though policy champions may have their own vested interests in policy processes, values and agendas [27,33]. Although a number of individuals championed the mental health cause, they often lacked power to make changes.

Greater involvement of under-represented policy actors in policy development, particularly non-health sectors, civil society and traditional healers, could improve the implementation of mental health policies, through better ownership of the policy, and use of existing community resources.

An interesting phenomenon in developing countries, particularly in Africa, is the role of external development partners, who through financial and technical assistance can influence policymaking by bringing an issue onto the agenda for action, as illustrated by the publication of the 2001 World Health Report [2]. However, these external partners played a limited role in influencing and supporting mental health policy processes. This may reflect the lack of interest by the international community, possibly due to other competing priorities such as MDGs including HIV/AIDS as well as a lack of targeted international funding towards mental health, compared to most communicable diseases.

Effective policy implementation is dependent on various factors, including the degree to which policies are translated into strategic and operational plans and the wider involvement of implementers during policy development. This suggests that implementation and possible challenges should be considered more fully at the policy development stage, to ensure feasibility.

Different resources are required for all stages of mental health policy processes. For example, policy *development *requires technical expertise and skills, timely and quality evidence, and finance to support organising of policymaking activities including involvement of stakeholders. The institutional resources within the health system are particularly important for policy *implementation *and include mental health staff and health managers, with appropriate numbers and competencies. Support systems and skills including budgeting, accounting, human resources management can be equally important resources to provide an enabling environment for mental health policy implementation [34].

Lastly, none of the above factors operate in isolation and various combinations of inter-relationships are possible with effects on mental health policy processes. For example, limited resources can determine the degree of consultation in health policy processes or availability and better quality evidence may empower civil society.

## Conclusions

Mental health policy processes in all four countries were inadequate, leading to either weak or non-existent policies, with an impact on mental health services. This is due to a number of factors set out in the framework related to the wider context, roles and characteristics of different policy actors and nature of mental health policy processes.

There is a need for greater effort to strengthen mental health policy processes in these and other African countries. In particular, there is a need to support the development and empowerment of CSOs, user groups and media organizations to raise the profile of mental health in national priorities and to reduce stigma regarding mental illness. As mental health is a multi-sectoral issue, this calls for greater involvement of different stakeholders in mental health policy process. Mental health policy processes are a neglected area of research and further work is needed to generate knowledge and test the applicability of the above findings and conclusions in other contexts.

## Competing interests

The authors declare that they have no competing interests.

## Authors' contributions

MO, AG, PB, TM and CL - participated in the conception and design of the study; analysis and interpretation of the data; and drafting the paper as well as revising it critically for substantial intellectual content.

AF and FG - participated in the conception and design of the study and revising the paper critically for substantial intellectual content.

JM and AO - participated in the data collection and analysis and revising the paper critically for substantial intellectual content. All authors read and approved the final manuscript.

MHaPP Research Programme Consortium - is a Research Programme Consortium (RPC) funded by the UK Department for International Development (DfID). Members of the Consortium participated in data collection, analysis and producing country reports.
